# Pressure generated at the instant of impact between a liquid droplet and solid surface

**DOI:** 10.1098/rsos.181101

**Published:** 2018-12-12

**Authors:** Y. Tatekura, M. Watanabe, K. Kobayashi, T. Sanada

**Affiliations:** 1 Graduate School of Engineering, Hokkaido University, Kita 13, Nishi 8, Kita-ku, Sapporo, Japan; 2 Graduate School of Engineering, Shizuoka University, 3-5-1 Johoku, Naka-ku, Hamamatsu, Japan

**Keywords:** droplet impact, impact pressure, water-hammer pressure, shock wave, shape factor

## Abstract

The prime objective of this study is to answer the question: *How large is the pressure developed at the instant of a spherical liquid droplet impact on a solid surface?* Engel first proposed that the maximum pressure rise generated by a spherical liquid droplet impact on a solid surface is different from the one-dimensional water-hammer pressure by a spherical shape factor (Engel 1955 *J. Res. Natl Bur. Stand.* **55**(5), 281–298). Many researchers have since proposed various factors to accurately predict the maximum pressure rise. We numerically found that the maximum pressure rise can be predicted by the combination of water-hammer theory and the shock relation; then, we analytically extended Engel’s elastic impact model, by realizing that the progression speed of the contact between the gas–liquid interface and the solid surface is much faster than the compression wavefront propagation speed at the instant of the impact. We successfully correct Engel’s theory so that it can accurately provide the maximum pressure rise at the instant of impact between a spherical liquid droplet and solid surface, that is, no shape factor appears in the theory.

## 1. Introduction

An understanding of liquid droplet impact onto a rigid solid surface is needed in a number of technological situations such as cleaning of surfaces, spray coating, spray cooling and ink-jet printing. Important practical situations are the erosion of blades in a steam turbine, in the wet steam erosion of forward-facing components on an aircraft, and erosion of hydraulic machinery including ship propellers [1–5]. When water drops impact a surface above a threshold velocity, they can eventually lead to erosion [6]. Liquid droplet impact is also one of the mechanisms leading to wall thinning, which causes rupture of the piping and unplanned outages of pressurized water reactors and boiled water reactors [7]. In order to determine the threshold velocity for the liquid droplet impact damage, a detailed knowledge of the liquid droplet impact process is required [4,8]. Obreschkow *et al.* [9] summarized the most important mechanisms of erosion that are known to occur during the impact of a liquid drop. A rigorous evaluation of the impact pressure generated on the solid surface at the instant of impact is essential for studying the very early stage of liquid droplet impact.

For the accurate prediction of the maximum pressure generated at the instant of impact, a large number of models have been proposed. Engel [6] estimated the maximum pressure rise Δ*P*_E_ from the atmosphere developed after a spherical liquid drop impact on a solid surface as

1.1 $$\Delta P_{E}=\frac{1}{2}\alpha_{E}\rho_{0}c_{0}V,$$

where *V* is the impact velocity, and *ρ*_0_ and *c*_0_ are the density and speed of sound, respectively, in the undisturbed liquid before the impact. The factor of $\frac{1}{2}$ is due to the ‘so-called’ spherical shape of the drop. It was found that the coefficient *α*_E_ is 0.4 using the Schlieren patterns produced by the collision of a water droplet with a glass plate. Later, Engel [10] proposed a general form of *α*_E_. The factor proposed by Engel became a controversial subject in droplet impact thereafter.

Jenkins & Booker [1] observed the impingement of water droplets, nominally of 2 mm diameter and roughly of spherical shape on a surface, moving at speeds ranging from 91 to 114 m s^−1^. They found that their result agrees with the so-called classical water-hammer pressure rise in water Δ*P*_w_, which is based on the relation

1.2 $$\Delta P_{w}=\rho_{0}c_{0}V.$$

Note that these studies [1,6,10] did not directly measure the pressure; instead, they evaluated the pressure using the experimentally obtained velocity. Rochester [11] and Rochester & Brunton [12] observed the impact pressure of a 5.0 mm diameter cylindrical water droplet when it was struck by a bullet at 100 m s^−1^. The pressure rise at instant of the impact is approximately 0.7*ρ*_0_*c*_0_*V*. Further, Rochester determined that the pressure is reduced at the centre of impact because the surface or the bullet deforms slightly during impact. The implication of this is that the pressure rises in the very early stage of impact against a solid surface are approximately 1.4 times those measured in the experiments. Therefore, he concluded that in an impact against a solid surface, these pressures become *ρ*_0_*c*_0_*V*. Bourne [13] evaluated the impact response of polymethylmethacrylate (PMMA) by measuring pressure in the region beneath the impacting liquid droplet with a radius of 2 mm by using polyvinylidene fluoride gauges at an impact velocity of 600 m s^−1^. He found that the initial impact pressure was approximately 0.8 GPa, which was just below the Hugoniot elastic limit for PMMA.

Heymann [14] introduced a two-dimensional model that represents a thin parallel slice taken vertically through the impacting drop. He inferred that the pressure rises are virtually uniform and equal to

1.3 $$\Delta P_{s}=\rho_{0}sV,$$

at the first instant of contact for the impact of a liquid droplet, where *s* in equation (1.3) is the compression wavefront speed [15] with a pressure that increases by Δ*P*_s_ with respect to the one in the undisturbed liquid pressure. His inference that the pressure rise at the instant of the impact for a liquid drop can be predicted by equation (1.3) is far from obvious. It is this inference that we primarily discuss in detail throughout this paper. Lesser [16] also claimed that the impact pressure at the instant of the impact on the centre can be estimated from the shock relation. Later, Field [17] explained that for the impact of a liquid droplet on a solid surface, the pressure rise on the central axis at the instant of impact is estimated by equation (1.3). Korobkin [18,19] generalized Lesser’s result to the whole region behind the shock front in his analysis on the impact by a flat plate on a circular liquid drop.

The numerical analysis conducted by Hwang & Hammitt [20] explored a new aspect of the study of liquid droplet impact. They investigated the impact process of cylindrical, spherical and conical water droplets on a solid plane surface using numerical methods. They found that the impact pressure rise reaches a peak of 0.7*ρ*_0_*c*_0_*V*. Haller [21] and Haller *et al.* [22] also numerically investigated the fluid dynamics of high speed (500 m s^−1^) small size (200 µm in diameter) droplet impact on a solid surface using a high-resolution axisymmetric solver for the Euler equations. Their numerical results support Lesser’s assumption.

These studies show that extensive efforts have been made toward the understanding of the pressure generated at droplet impact; however, the maximum pressure rise at the impact on the central axis has yet to be indisputably determined. Recent various numerical analyses indeed provide a variety of results. Sanada *et al.* [23] carried out a numerical simulation of an axisymmetric spherical liquid droplet impact on a solid surface, using the ghost fluid method [24]. They found that the maximum pressure rise of the point closest to the solid surface can be well predicted by equation (1.2). Xiong *et al.* [7] found that the maximum average impact pressure given by their simulation agrees well with the Heymann correlation pressures [14] using the moving particle semi-implicit method. Sanada *et al.* [25] and Kondo & Ando [26] numerically studied axisymmetric and cylindrical droplet impact on a solid surface, respectively, using the shock-interface capturing scheme [27]. Their results support Engel [6], i.e. equation (1.1). Hsu *et al.* [28] also supported equation (1.1) as an empirical formulation that can predict the maximum pressure rise for the impingement of a spherical liquid drop.

Note that pressure impulse theory, which Cooker & Peregrine [29] and Philippi *et al.* [30] used, and most of classical work on the fluid–solid impact, including the seminal paper by Wagner [31], Howison *et al.* [32] and Scolan & Korobkin [33], were carried out under the assumption of an ideal and incompressible liquid. However, the compressibility of the liquid plays the most significant role in the prediction of the maximum pressure rise. The most general methods to investigate the pressure generated at the instant of impact between a liquid droplet and solid surface are those that appropriately take the liquid compressibility into consideration.

The prime objective of this study is to answer the question: *How large is the pressure developed at the instant of a liquid droplet impact on a solid surface?* by shedding light on the shape factor that is still controversial. To answer this question, we consider the impact of a spherical liquid droplet on a solid surface under the assumption that liquid droplet keeps its spherical shape at the instant of the impact, although it is well known that a liquid droplet impacting on a solid surface deforms before the liquid droplet makes contact with the surface [34–36] or entraps a small air bubble under its centre under atmospheric conditions [37,38]. The reason is that we primarily discuss the role of the spherical shape factor introduced by Engel [6], who also used the assumption that the liquid droplet keeps its spherical shape at the instant of the impact, in the central impact pressure rise. We numerically estimate the central impact pressure (defined here as the pressure developed at the instant of the impact on the contact point on the axis of symmetry) by solving two-dimensional axisymmetric compressible Euler equations. We then extend Engel’s elastic impact model [6] taking the limit of the time from impact for the pressure to reach its maximum to 0, by realizing that the progression speed of the contact between the gas–liquid interface and the solid surface is much faster than the compression wavefront propagation speed at the instant of the impact. We correct Engel’s elastic impact model to find that the factor *α*_E_/2 appearing in equation (1.1) converges to 1 at the instant of the impact.

## 2. Previous models for one-dimensional analyses of impact pressure rise

As stated in the previous section, the water-hammer theory has been often used in the analysis of liquid droplet impact pressure rise. We also contrast the liquid droplet impact pressure with the water-hammer pressure rise in later sections; hence, in this section, we briefly review a couple of previous one-dimensional models used in the estimation of impact pressure.

A sudden halt of flow in a pipe is considered to be equivalent to a liquid droplet impact on a solid surface. Cook [39] was one of the first to realize that the pressure generated on an element of a surface at its first encounter with water moving at a finite velocity would be given by the water-hammer pressure rise. Gardner [40] and de Haller [41] extended this analysis by accounting for the compressibility of the solid.

**Figure 1.**
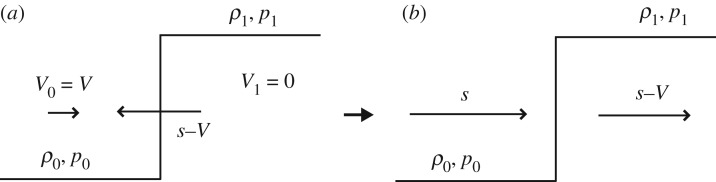
Water-hammer pressure rise in (*a*) a stationary coordinate system, and (*b*) a moving coordinate system with a speed of (*s* − *V*), where *s* is the compression wavefront speed in a quiescent liquid and *ρ*_0_, *p*_0_, and *V* are density, pressure and speed of the uniform flow of the undisturbed liquid, respectively. A high pressure builds and propagates upstream (to the left in (*a*)) with a speed of (*s* − *V*). *ρ*_1_ and *p*_1_ are the density and pressure of the liquid that the pressure wave has reached, respectively.

Suppose a uniform flow of a liquid moving with a speed of *V* in a pipe to the right in figure 1*a*. When the pipe is suddenly closed at the valve, a high pressure builds and propagates upstream (to the left in figure 1*a*) with a speed of (*s* − *V*), where *s* is the compression wavefront speed in a quiescent liquid. Figure 1*a* can be redrawn as figure 1*b* using the coordinate system moving with a speed of (*s* − *V*).

**Figure 2.**
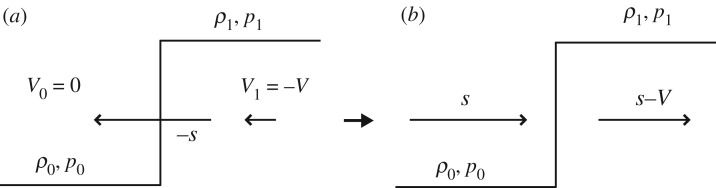
Shock relation in (*a*) a stationary coordinate system, and (*b*) a moving coordinate system with the compression wavefront speed *s*. The shock front is generated with a speed of *s* moving into a quiescent medium with density *ρ*_0_ and pressure *p*_0_ as shown in (*a*), where *ρ*_1_ and *p*_1_ are the fluid density and pressure of the liquid behind the compression wavefront, respectively.

Heymann [14], on the other hand, used the so-called shock relation to estimate the pressure rise developed at a liquid droplet on a solid surface. He considered that a moving solid plate with a velocity *V* collides with a stationary liquid droplet. Then, a shock front is generated with a speed of *s* moving into a quiescent medium, as shown in figure 2*a*. The liquid velocity induced behind the shock front is the same as the plate velocity *V*. Figure 2*a* can be redrawn as figure 2*b* using the coordinate system moving with a velocity of *s*.

Note that both figures 1*b* and 2*b* are identical; hence, the conservation equations of mass, momentum and energy for both cases should be identical and are, respectively, written as

2.1 $$\rho_{0}s=\rho_{1}(s-V),$$

2.2 $$p_{0}+\rho_{0}s^{2}=p_{1}+\rho_{1}{\left(s-V\right)}^{2}=p_{1}+\rho_{0}s(s-V)$$

and

2.3 $$p_{0}s+\rho_{0}s\left[\frac{s^{2}}{2}+e_{0}\right]=p_{1}(s-V)+\rho_{1}(s-V)\left[\frac{{\left(s-V\right)}^{2}}{2}+e_{1}\right].$$

Equations (2.1) and (2.2) lead to the following expression of pressure rise Δ*P*_s_:

1.3 $$\Delta P_{s}=p_{1}-p_{0}=\rho_{0}sV.$$

The classical water-hammer pressure rise (1.2) can be obtained by simplifying equation (1.3) using the commonly used assumption that the compression wavefront speed can be replaced with *c*_0_, the speed of sound in an undisturbed liquid:

2.4 $$s=c_{0}.$$

Further review on this topic can be found in Brunton & Rochester [42] and Lesser & Field [43].

We now consider *s* as a variable instead of a constant to more accurately predict the maximum pressure rise. We need an additional equation to close the equation set. We examine two different equations for properly evaluating *s*. The first equation was empirically developed by Heymann [15] and is the approximate shock velocity–particle velocity relationship:

2.5 $$s=c_{0}\left[1+k\left(\frac{V}{c_{0}}\right)\right]=c_{0}(1+kM_{0}),$$

where

2.6 $$M_{0}=\frac{V}{c_{0}}$$

is the ‘impact Mach number’ and *k* is a dimensionless constant. Heymann [14] obtained the impact pressure rise using the approximate shock velocity–particle velocity relationship Δ*P*_H_:

2.7 $$\Delta P_{H}=\rho_{0}c_{0}V(1+kM_{0}).$$

Heymann [15] found that the value *k* = 2 fits the data for water up to about *M*_0_ = 1.2. Substituting the values *c*_0_ = 1497 m s^−1^ and *V* = 304.8 m s^−1^ used by Engel [44] into equation (2.7), an impact pressure of 1.41*ρ*_0_*c*_0_*V* can be obtained with the assumption of a rigid solid impact plate.

The second equation is the stiffened-gas equation of state proposed by Harlow & Amsden [45]:

2.8 p=(γ−1)ρe−γΠ,

where *e* is the internal energy per unit mass, *γ* and *Π* are constants (*γ* = 1.4 and *Π* = 0 for air; *γ* = 4.4 and *Π* = 6.0 × 10^8^ for water). Equation (2.8) is widely used for computational analyses to investigate compressible gas–liquid two-phase flow [46–48]. Saurel & Abgrall [46] reported that it is possible to describe compressible liquid under high pressure with reasonable accuracy using the stiffened-gas equation of state, and that each phase of the flows involving various materials may be described by an equation of state of this type. We also implemented equation (2.8) in our numerical analysis, as explained in the next section.

Equations (2.1), (2.2) and (2.3) with the stiffened-gas equation of state (2.8) give the speed of sound for the stiffened-gas [49]:

2.9 $$s=c_{o}\left[\frac{(\gamma +1)M_{0}}{4}+\sqrt{1+\frac{{\left(\gamma +1\right)}^{2}M_{0}^{2}}{16}}\right].$$

Using equation (2.9), we obtain the impact pressure rise with the use of stiffened-gas equation of state Δ*P*_sg_:

2.10 $$\Delta P_{\mathrm{sg}}=\rho_{o}c_{o}V\left[\frac{(\gamma +1)M_{0}}{4}+\sqrt{1+\frac{{\left(\gamma +1\right)}^{2}M_{0}^{2}}{16}}\right].\;$$

We found in this section that the one-dimensional liquid impact pressure rise Δ*P*_s_ on a solid surface can be formally written

2.11 $$\Delta P_{s}=\rho_{0}sV,$$

regardless of the choice of the model. All these models were derived from the same conservation equations; however, different equations for the compression wavefront speeds were used. We refer to these pressure rises, Δ*P*_w_: equation (1.2), Δ*P*_H_: equation (2.7) and Δ*P*_sg_: equation (2.10), as the classical water-hammer impact pressure rise, Heymann impact pressure rise and stiffened-gas impact pressure rise, respectively, in the following discussion.

## 3. Numerical evaluation of the pressure developed immediately after the liquid droplet impact

### 3.1. Numerical method

We numerically investigated the liquid droplet impact pressure rise on a solid surface. We analysed the pressure generation at droplet impact by solving the two-dimensional axisymmetric compressible Euler equations [22,50]. To close the Euler equations, we implemented the stiffened-gas equation of state (2.8) in our numerical analysis; hence, we compare the pressure rise due to the liquid droplet impact obtained by numerical analysis with the stiffened-gas pressure rise Δ*P*_sg_ (2.10). We solved the flow field using the level-set method [51,52] combined with the ghost fluid method [24,53,54]. We used a finite difference method for discretization of the system. The third-order TVD Runge–Kutta scheme was employed to march the equations forward in time, and the third-order ENO-LLF scheme was employed to calculate the convection term [55,56]. The third-order TVD Runge–Kutta scheme and fifth order WENO scheme [57] was employed to advance the level-set function *ψ*, where sets of *ψ* = 0, *ψ* < 0 and *ψ* > 0 represent the gas–liquid interface, one fluid and the other fluid, respectively. We calculated the physical properties only in the liquid phase with constant gas pressure. Haller *et al.* [22] carried out a numerical simulation of droplet impact on a solid surface by solving flow fields of both liquid and gas phases. Their results succeeded to reproduce the theoretical results by Lesser [16] who did not consider the dynamics of the gas phase. Hence, we can neglect the contribution of gas that was also neglected by the theoretical models for the purpose of this study.

We introduced the assumption that the portion of the droplet that has not collided remains spherical. This should hold in the computation partly because we mainly investigate the pressure development immediately after the droplet impact, where the deformation may be insignificant, and partly because we discuss the legitimacy of the theoretical models by comparing them with the numerical results. Therefore, the contour line of *ψ* = 0 is enforced to maintain a circle whose centre moves with impact velocity *V* in *z* > 0.

Impact velocity *V* was set to 100 m s^−1^ because this order of magnitude of impact velocity can be typically observed in cleaning technologies for semiconductor device processes [23,50,58,59], in single water droplet impact experiments [11,60,61] and in numerical simulations of liquid droplet impact [9,26]. Note that the difference between *c*_0_ and *s* is hardly noticeable if the value of *V* is much smaller, and the stiffened-gas equation of state (2.8) is not applicable if the value of *V* is much larger.

**Figure 3.**
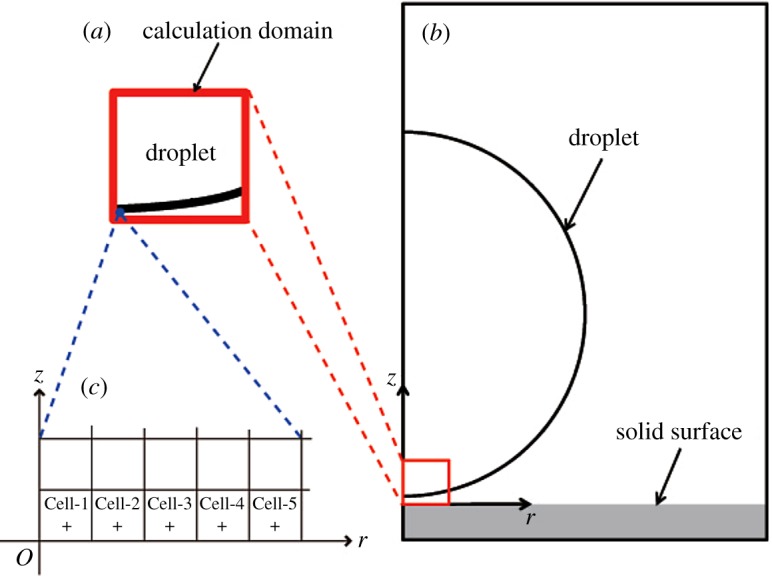
Calculation domain. The square shown in (*a*) is the computational domain used in this study. This computational domain is a portion of the configuration of the liquid droplet impact on the solid surface shown in (*b*). The length of the side of the square is *R*/4, where *R* is the droplet radius at the initial state and was set to 1 mm. The size of the square calculation cell Δ*r* is non-dimensionalized: $\Delta r^{*}=\Delta r/R$ with the range of $2^{-15}\leq \Delta r^{*}\leq 2^{-4}$. The initial pressure was set to 0.1013 MPa. The initial density and speed of sound were set to 1000 kg m^−3^ and 1625 m s^−1^ for the liquid.

We calculated the pressure of the fluid in the cell contacting the solid surface. We then identified this pressure using the pressure on the solid surface. We refer to these cells adjacent to the solid surface as the surface cells. We define Cell-1 to be the left-most surface cell contacting with the *z*-axis, as shown in figure 3*c*. We consider the gas–liquid interface to impact on the solid surface when the fluid in the surface cell that is adjacent to the point turns from gas to liquid, i.e. when the interface crosses the centre of the surface cell. We also consider the droplet to impact on the solid wall when the gas–liquid interface impacts on the solid surface in Cell-1. The central pressure is defined as the pressure in Cell-1. We discuss the temporal change of the central pressure after the droplet impacts on the solid surface. We also investigated the pressures in other surface cells, particularly Cell-2, Cell-3, Cell-4 and Cell-5, as shown in figure 3*c*.

### 3.2. Estimation of the central pressure

**Figure 4.**
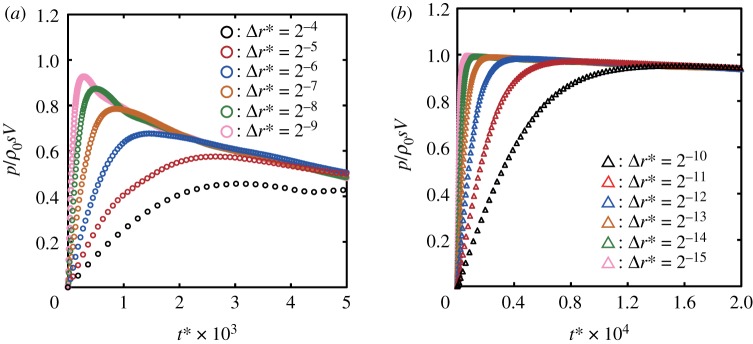
Convergence of the maximum central pressure obtained by numerical analysis as the non-dimensional calculation cell size $\Delta r^{*}$ decreases: (*a*) $2^{-9}\leq \Delta r^{*}\leq 2^{-4}$ and (*b*) $2^{-15}\leq \Delta r^{*}\leq 2^{-10}$. The calculated pressure rise was normalized by the stiffened-gas impact pressure rise Δ*P*_sg_ defined by equation (2.10). Note that the scale of the abscissa of (*b*) is 25 times greater than that of (*a*).

We now examine the maximum central pressure rise Δ*P*_max_ at the instant of impact. Figure 4 shows the temporal changes of the pressure in Cell-1 calculated with various cell sizes, where the non-dimensionalized time $t^{*}$ was defined by $t^{*}=Vt/R$. We use the following expression for the maximum central pressure rise:

3.1 $$\Delta P_{\max}=\alpha \Delta P_{\mathrm{sg}}=\alpha \rho_{0}sV,$$

where *α* is defined as the ratio of the calculated maximum central pressure rise to the stiffened-gas impact pressure rise, and *s* is defined by equation (2.9). Throughout this paper, we use the constant speed of compression wavefront *s* = 1760 m s^−1^, which is obtained by substituting *V* = 100 m s^−1^ and *c*_0_ = 1625 m s^−1^ in equation (2.9). It is a remarkable finding that *α* depends on $\Delta r^{*}$; *α* reaches 0.994 in the case of $\Delta r^{*}=2^{-15}$, while it is 0.455 in the case of $\Delta r^{*}=2^{-4}$, as shown in figure 4. Surprisingly, the variation in the value of *α* is more than double.

**Figure 5.**
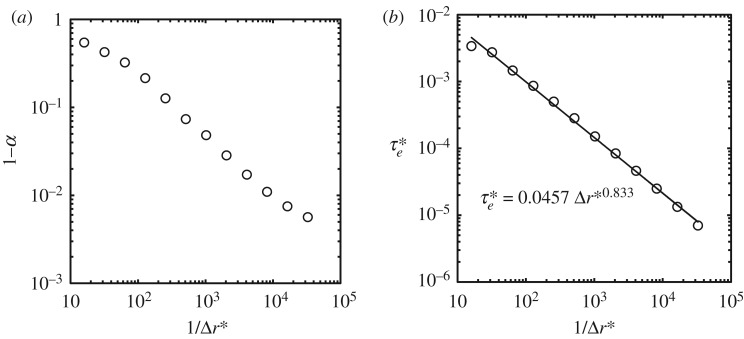
(*a*) The dependency of *α* (the ratio of the calculated maximum central pressure rise to the the stiffened-gas impact pressure rise as in equation (2.10)) on $\Delta r^{*}$. (1 − *α*) (in log-scale) is plotted on the ordinate and $\Delta r^{*}$ (in log-scale) is plotted on the abscissa. (*b*) The dependency of $\tau^{*}$ (non-dimensional elapsed time from the instant of impact until the pressure reaches the maximum) on $\Delta r^{*}$. $\tau^{*}$ (in log-scale) is plotted on the ordinate and $\Delta r^{*}$ (in log-scale) is plotted on the abscissa The regression line shows that $\tau_{e}^{*}$ is approximately proportional to $\Delta r^{*}$.

We evaluate *α* for each $\Delta r^{*}$. Figure 5*a* shows that *α* monotonically converges to 1 when $\Delta r^{*}$ decreases. Therefore, we conclude that the maximum central pressure rise at the instant of impact Δ*P*_max_ is given by

3.2 $$\Delta P_{\max}=\Delta P_{\mathrm{sg}}=\rho_{0}sV.$$

Note that the results of the numerical simulation provide the maximum central pressure rise that can be expressed by equation (3.2) under very restricted conditions, where $\Delta r^{*}$ is sufficiently small. For instance, we should choose a $\Delta r^{*}$ that is less than 2^−14^ to obtain an *α* greater than 0.99. We found that significantly small calculation cells are required to resolve the magnitude of the pressure at the instant of impact.

We also found in figure 4 that *τ*_*e*_, the elapsed time for the pressure to reach its maximum from the instant of impact, decreases as $\Delta r^{*}$ decreases. Non-dimensionalized elapsed time $\tau_{e}^{*}\;(=\tau_{e}V/R)$ is plotted in figure 5*b*. Note that the impact pressure attains the maximum pressure because of the elastic compression of the fluid in the cell; hence, $\tau_{e}^{*}$ decreases as $\Delta r^{*}$ decreases. Therefore, in the limit taken as $\Delta r^{*}$ approaches 0, the elapsed time to reach the maximum central pressure converges to 0.

### 3.3. Maximum central pressure dependency on calculation cell size

**Figure 6.**
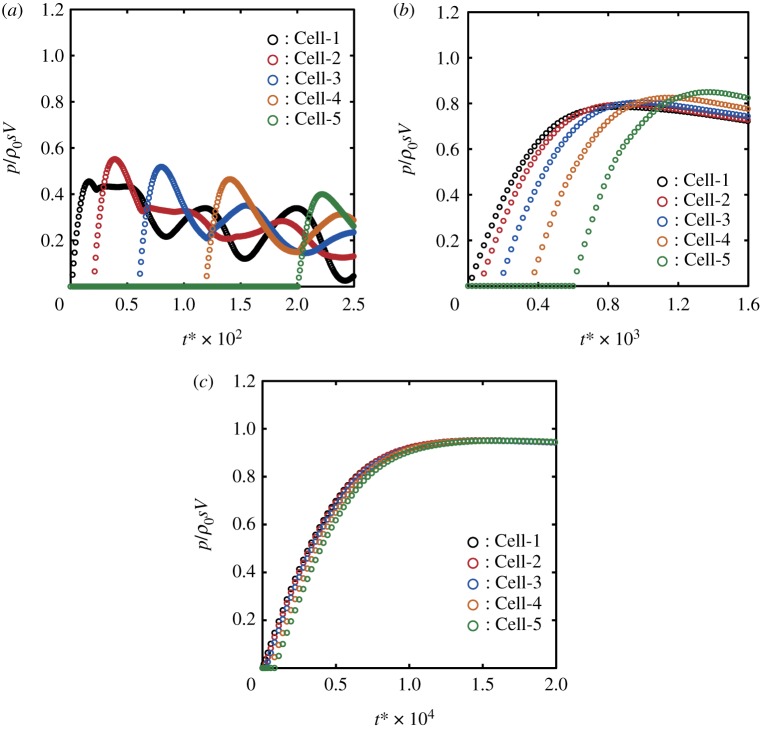
Pressure developments in the first five surface cells for the cases of (*a*) $\Delta r^{*}=2^{-4}$, (*b*) $\Delta r^{*}=2^{-7}$ and (*c*) $\Delta r^{*}=2^{-10}$. (*a*) The pressure in Cell-2 begins to rise only after the pressure in Cell-1 has reached the maximum; then, *α* is 0.455. (*b*) The pressure in Cell-2 begins to rise when the pressure in Cell-1 is still increasing; then, *α* is much greater than 0.455 but much less than 1.0. (*c*) The pressure in Cell-2 begins to rise immediately after the pressure in Cell-1 has begun to rise; then, *α* is very close to 1.

We found that *α* can converge to 1 as long as $\Delta r^{*}$ is sufficiently small in §3.2; however, *α* can reach at most 0.455 when $\Delta r^{*}=2^{-4}.$ We now discuss the dependency of *α* on $\Delta r^{*}$. For a large cell size ($\Delta r^{*}=2^{-4}$ in figure 6*a*), the pressure in Cell-2 begins to rise only after the pressure in Cell-1 reaches the maximum; that is, Cell-2, Cell-3, Cell-4 and Cell-5 are filled with gas while the pressure in Cell-1 is increasing; then, *α* is 0.455. For a medium cell size ($\Delta r^{*}=2^{-7}$ in figure 6*b*), the pressure in Cell-2 begins to rise when the pressure in Cell-1 is still increasing; that is, the fluid in Cell-2 turns from gas to liquid while the pressure in Cell-1 increases; then, *α* is much greater than 0.455 but much less than 1.0. For a small cell size ($\Delta r^{*}=2^{-10}$ in figure 6*c*), the pressure in Cell-2 begins to rise immediately after the pressure in Cell-1 has begun to rise; that is, Cell-2 is almost filled with liquid while the pressure in Cell-1 is increasing; then, *α* is very close to 1. These results suggest that the status of Cell-2, i.e. either liquid or gas, should greatly affect the central maximum pressure.

**Figure 7.**
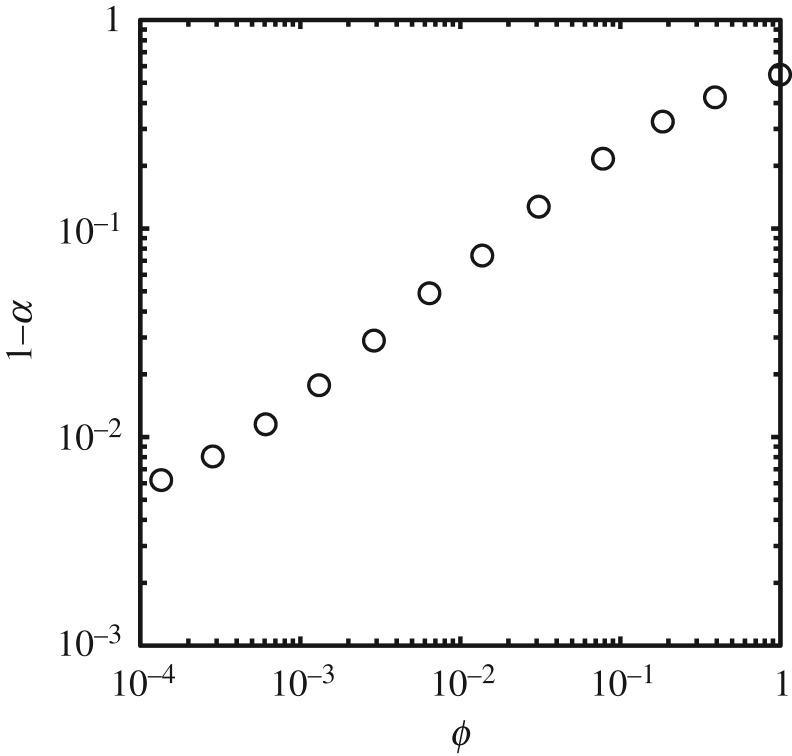
The ratio of time *ϕ*, $\phi =\tau_{2}^{*}/\tau_{1}^{*}$, is plotted on the abscissa (in log-scale) with (1 − *α*) on the ordinate (in log-scale). The values of *ϕ* for $\Delta r^{*}$ of 2^−4^, 2^−7^ and 2^−10^ are 1.00, 7.86 × 10^−2^ and 6.46 × 10^−3^, respectively. Here, *α* monotonically increases as *ϕ* decreases. This indicates that the time duration for which the fluid in Cell-2 is liquid while the pressure in Cell-1 is increasing completely dominates the value of *α*.

We now introduce the ratio of time *ϕ*:

3.3 $$\phi =\frac{\tau_{2}^{*}}{\tau_{1}^{*}},$$

where $\tau_{1}^{*}$ is the non-dimensional time which it takes for the pressure in Cell-1 to rise from *p*_0_ to *P*_max_, and $\tau_{2}^{*}$ is the non-dimensional duration over which the fluid in Cell-2 is gas for $\tau_{1}^{*}$. Figure 7 shows that *α* monotonically increases as *ϕ* decreases. We can see that the time duration for which the fluid in Cell-2 is liquid while the pressure in Cell-1 is increasing totally dominates the value of *α*.

When the fluid in Cell-2 turns to liquid immediately after the pressure rises in Cell-1, the liquid in Cell-1 is confined to the space bounded by the liquid in Cell-2, for which the pressure is about the same as that of Cell-1; hence, the liquid in Cell-1 can be compressed primarily in the direction normal to the solid surface. This compression can be thought of as the one-dimensional liquid compression by which the pressure generated can be predicted well by equation (2.10). Therefore, *α* is essentially equal to 1 when *ϕ* is sufficiently close to 0.

When the fluid in Cell-2 remains gas after the pressure rises in Cell-1, the liquid in Cell-1 is confined to the space bounded by the gas in Cell-2; hence, the liquid in Cell-1 is able not only to be compressed in the normal direction but also to be expanded in the direction parallel to the solid surface. In other words, the liquid in Cell-1 can flow in the direction parallel to the solid surface. As Bowden & Field [2] explained, *α* cannot reach 1 when the liquid in Cell-1 can flow. According to them, *α* can reach 1 when the liquid in Cell-1 cannot flow, that is, the flow can be assumed to be one-dimensional. Therefore, *α* is significantly smaller than 1 when *ϕ* is close to 1.

### 3.4. Impact of an interface with a finite curvature

According to the numerical analyses conducted by Sanada *et al.* [25] and Kondo & Ando [26], the pressure rise in liquid impact on a solid wall can be well predicted by using the Heymann impact pressure rise (equation (2.7)) when the impact can be considered as a one-dimensional impact, where the radius of curvature of the impacting interface is infinite, i.e. a plane interface. However, they also found that liquid droplet impact pressure rise on the same solid wall can be significantly reduced when the radius of curvature of the impacting interface is finite: they numerically reproduced a cylindrical droplet impact on a solid wall. The latter pressure was approximately 0.2 of the former pressure. Their results apparently supported Engel [6], i.e. equation (1.1). We now examine whether the liquid impact pressure rise can be reduced by the stiffened-gas pressure rise (equation (2.10)) when the interface that impacts on a solid surface has a finite radius of curvature using the numerical analysis.

We found that the impact pressure rise can reach the one-dimensional liquid impact pressure rise when $\Delta r^{*}$ is sufficiently small in our numerical simulation. We must carefully examine the possibility that the interface of the liquid droplet might be plane with an infinite radius of curvature when the liquid droplet impacts on a solid surface for sufficiently small $\Delta r^{*}$ because the plane interface impact might be equivalent to a one-dimensional impact, which leads to the stiffened-gas pressure rise (equation (2.10)). Therefore, we need to confirm whether the liquid droplet impacts a solid surface with a finite radius of curvature.

**Figure 8.**
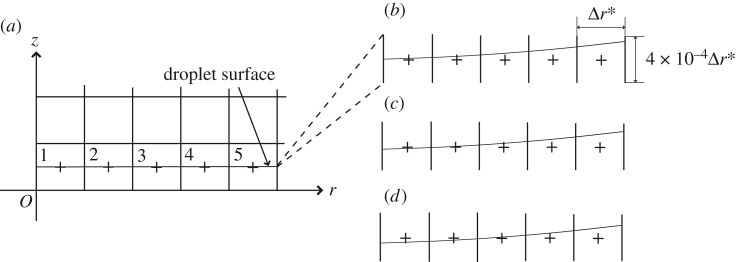
Impact of an interface with a finite curvature with a solid surface. No simultaneous interface impact occurs in any adjacent cells. (*a*) Location of the interface in Cell-1, Cell-2, Cell-3, Cell-4, and Cell-5, (*b*) $t^{*}/\Delta t^{*}=0$, (*c*) $t^{*}/\Delta t^{*}=1$, (*d*) $t^{*}/\Delta t^{*}=3$. Note that the ordinates in (*b*–*d*) are greatly magnified with respect to (*a*). The numerical calculation was carried out under the conditions that $\Delta r^{*}=2^{-15}$ and $\Delta t^{*}=3.73\times 10^{-10}$.

**Figure 9.**
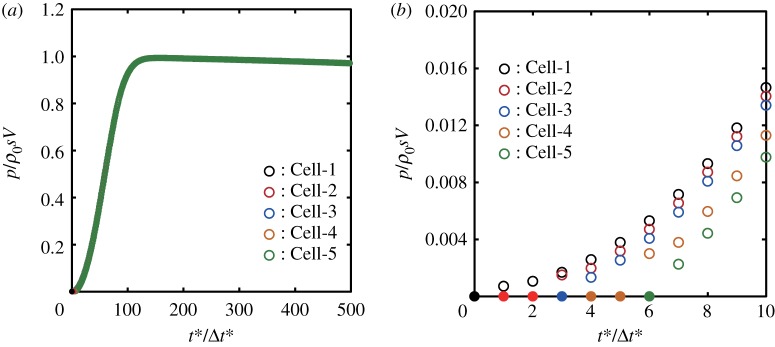
Pressure development at the first five surface cells at droplet impact: (*a*) pressure development; (*b*) enlarged view of (*a*) at a very early stage of the impact. The interface impacts on the solid surface in Cell-1 at $t^{*}/\Delta t^{*}=1$. The pressure of the liquid in Cell-1 then rises while the pressure of the liquid in the other cells remains unchanged. The shape of the interface at $t^{*}/\Delta t^{*}=1$ is drawn in figure 8*c*. The interface impacts on the solid surface in Cell-2 at $t^{*}/\Delta t^{*}=3$. The shape of the interface at $t^{*}/\Delta t^{*}=3$ is drawn in figure 8*d*. The interface impacts on the solid surface in Cell-3, Cell-4 and Cell-5 at $t^{*}/\Delta t^{*}=4$, 6 and 7, respectively.

When a gas–liquid interface impacting on a solid surface is a plane, the impacts of the interface simultaneously occur in adjacent multiple cells; hence, the impacts of the interface should not simultaneously occur in adjacent cells in figure 8 when the interface has a finite radius of curvature. Figure 9*a* shows the pressure development at the five surface cells in figure 8, although the pressures observed at different surface cells are identical. Figure 9*b* shows an enlarged view of the very early stage of droplet impact. Therefore, we conclude that we successfully reproduced the impact of a droplet which has a finite radius curvature with a solid surface.

We now consider the maximum central pressure rise. Figure 9*a* shows that the maximum pressure rises observed at the surface cells Cell-1 to Cell-5 shown in figure 8 are identical. We find that the factor *α* defined by equation (3.1) is 0.994 in figure 9. Therefore, we conclude that the maximum central pressure rise that develops when a droplet impacts a solid surface can be accurately predicted by equation (2.10), *even with a finite radius of curvature interface*.

To close this section, we add the following as the final remarks. It requires extremely high resolutions in both the spatial and temporal domains to obtain the numerical results that confirm that the maximum central pressure rise can be predicted by equation (2.10). We are convinced that insufficient resolutions in either the numerical or experimental studies have caused the confusion in determining the factor.

## 4. Equation for the central pressure resulting from a liquid droplet impact

We now return to the Engel pressure rise Δ*P*_E_:

1.1 $$\Delta P_{E}=\frac{1}{2}\alpha_{E}\rho_{0}c_{0}V.$$

Engel stated with regard to equation (1.1) that *this is the well-known water-hammer equation multiplied by factor *α*_E_/2, which comes from the spherical shape of the liquid droplet*; indeed, it has long been understood that the factor of $\frac{1}{2}$ is considered to be a consequence of the spherical shape of the drop [28]. However, our numerical analysis showed that the impact pressure rise due to the spherical liquid droplet can be predicted by equation (2.10), where no shape factor is required. In this section, we reconsider the origin of the factor of $\frac{1}{2}$ and then discuss the legitimacy of using equation (1.1) for predicting the maximum central pressure rise.

### 4.1. Maximum pressure rise proposed by Engel

**Figure 10.**
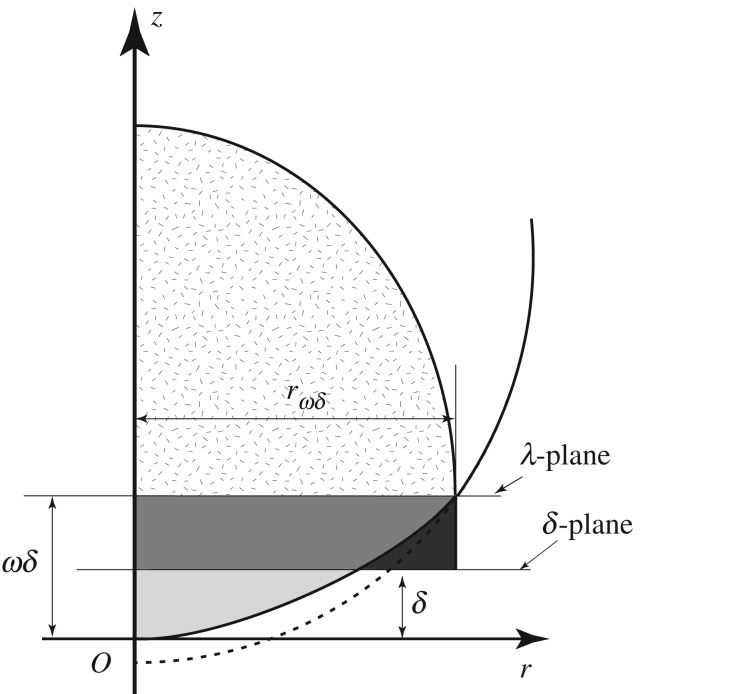
Cross section of the liquid droplet reproduced from fig. 14 in [6]. First, the impact of a droplet of radius *R* (dashed curve) with a solid plane surface forces the radius of curvature of the droplet in the vicinity of the impact to change instantaneously to *R*′ (solid curve), and a compression wavefront to propagate through the liquid droplet, which induces a velocity in the *z*-direction in the liquid (the compression process). Second, the moving solid plane surface of velocity *V* travels through the liquid whose average velocity may be written as *α*_E_*V* in the time interval Δ*t* to reach the *δ*-plane. The relative velocity of the moving solid plane surface to the liquid droplet therefore becomes (1 − *α*_E_)*V*; hence, the compressed cap, which is shown as the light grey region, with height *δ*, *δ* = (1 − *α*_E_)*V*Δ*t*, is displaced into a very thin cylinder of liquid that is in radial flow (the displacement process). The liquid cylinder with a base radius of *r*_*ωδ*_ formed between the *δ*-plane and the λ-plane has the same volume as that of the spherical cap whose base is on the λ-plane with a height of *ωδ*.

Engel [6] derived equation (1.1) for the maximum pressure rise Δ*P*_E_ that develops at the time Δ*t* after a liquid droplet impacts on a solid surface. We now briefly introduce the analysis by Engel. She considered that the liquid droplet impact consists of two sequential (not simultaneous) processes: a compression which is an instantaneous process and a subsequent displacement which takes a finite time Δ*t*, as shown in figure 10 drawn based on fig. 14 in [6]. Engel introduced a coefficient *α*_E_, which is an index of the fraction of velocity *V* that is imparted to the liquid molecules on average. She assumed that the coefficient *α*_E_ must be governed mainly by the extent of divergence of the compression wave as it spreads through the spherical droplet. She also assumed that the amount of divergence of the wave decreases and the value of the coefficient *α*_E_ approaches unity as the impact velocity increases.

To obtain the maximum pressure rise Δ*P* at time *t* = Δ*t*, Engel considered the total mass of liquid *m* that gains momentum during a *finite* time interval Δ*t*, and consequently all the force that acts to produce the momentum over time interval Δ*t*. Engel considered a separated pair of regions. First, she considered the mass *m*′ of the spherical cap, which gains a momentum that causes a radial flow. She defined the λ-plane to be the upper boundary of the radial flow and considered it to be an effective striking surface with which compression wavelets are initiated at the points of contact of the remaining liquid droplet. The distance between the *δ*- and λ-planes is (*ω* − 1)*δ* where *ω* ≥ 1. She noted that in the limit, the volume of a spherical cap is one-half the volume of a cylinder that has the same height and base area.

Engel then assumed that the first liquid of the droplet that encounters the moving solid plane surface during the time interval Δ*t* is displaced to form a thin cylinder the height of which is determined so that the volume of the spherical cap below the λ-plane before the displacement (the union of medium grey and light grey regions in figure 10) is equal to that of the cylinder (the union of medium grey and dark grey regions in figure 10); hence, *ω* is equal to 2. During time Δ*t*, which is the time needed for the moving solid plane surface with the relative velocity (1 − *α*_E_)*V* to move a distance *δ* through the droplet, the λ-plane moves a distance *ωδ*. Velocity $V_{\lambda }$ of the λ-plane is then given by

4.1 $$V_{\lambda }=\frac{\omega \delta }{\Delta t}=\omega (1-\alpha_{E})V.$$

The mass of the spherical cap (the union of medium grey and light grey regions in figure 10) whose base radius is *r*_*ωδ*_, is given by

4.2 $$m^{\prime }=\rho_{0}v_{m^{\prime }}=\frac{\omega \pi }{2}r_{\omega \delta }^{2}\delta ,$$

where *v*_*m*′_ is the volume of the spherical cap. Engel mentioned that although the mass of liquid *m*′ moves horizontally after time Δ*t*, it was given as a velocity in the collision direction *z* during this time interval because it was traversed by the compression wave.

Engel next considered the region (with mass *m*″) of the liquid droplet that has been traversed by the compression wave during the time interval Δ*t*, which is shown as a hatched region in figure 10; then, she assumed that the average velocity of the liquid in this region may be written as *α*_E_*V*. Suppose that the radius of the circular intersection between the λ-plane and the surface of the spherical cap is *r*. The compression wavelet can be generated on this circular intersection with 0 ≤ *z* ≤ *ωδ* when the λ-plane hits the surface of the spherical cap as shown in figure 10. The λ-plane is relatively stationary with respect to the droplet in the compression process because it keeps contact with a point at the first instant of impact, while it moves a distance *ωδ* in the displacement process over time Δ*t*. The compression wave consists of each compression wavelet, which travels with the velocity *s*. Engel assumed that the compression wavelet travels only in the *z*-direction. Recall that *z* = *ωδ* at *r* = *r*_*ωδ*_; hence,

4.3 $$r_{\omega \delta }^{2}=2R^{\prime }\omega \delta -{\left(\omega \delta \right)}^{2}≃2R^{\prime }\omega \delta .$$

Let *z*′ be the distance that the compression wavelet that originated from the circular intersection with radius *r* travels above the λ-plane at *z* = *ωδ*. The distance Δ*z* between the circular intersection and the λ-plane can be written as

4.4 $$\Delta z=\omega \delta -z=\omega \delta -R+\sqrt{R^{2}-r^{2}}.$$

Suppose that the λ-plane moves by Δ*z* to reach *z* = *ωδ* within time Δ*τ* after the compression wavelet was generated:

4.5 $$\Delta \tau =\frac{\Delta z}{\omega (1-\alpha_{E})V},$$

then,

4.6 $$z^{\prime }=\Delta \tau s-\Delta z=\left(\frac{s}{\omega (1-\alpha_{E})V}-1\right)\Delta z=\left(\frac{s}{V_{\lambda }}-1\right)\Delta z=\beta \Delta z,$$

for Δ*z*, 0 ≤ Δ*z* ≤ 2*δ*, where *β* is defined as $\beta =s/V_{\lambda }-1$. Note that the locus of points *z*′ gives the envelope of the compression wavelets.

Next, Engel evaluated the volume of the region *v*_*m*″_ that has been traversed by the compression wave during the time interval Δ*t*, which is indicated by the hatched region in figure 10. Volume *v*_*m*″_ is that of the body of revolution bounded by the envelope and λ-plane at *z* = *ωδ*. Then,

4.7 $$\begin{array}{rl} v_{m^{\prime \prime }} & =\pi \int_{0}^{\omega \beta \delta }r^{2}dz^{\prime }={\left[-\frac{z^{\prime 3}}{3\beta^{2}}+\frac{\left(\omega \delta -R\right)}{\beta }z^{\prime 2}-(\omega^{2}\delta^{2}-2\omega \delta R)z^{\prime }\right]}_{z^{\prime }=0}^{\omega \beta \delta }\\ & =\omega^{2}\beta \pi \delta^{2}\left(R-\frac{\omega }{3}\delta \right)≃\frac{\omega }{2}\beta \pi r_{\omega \delta }^{2}\delta . \end{array}$$

Engel estimated that the average rate of change of momentum of liquid in volume (*v*_*m*′_ + *v*_*m*″_) is (*m*′ + *m*″)*α*_E_*V*/Δ*t*. She also assumed that all of the force that has acted to produce the liquid momentum is written as $\pi r_{\omega \delta }^{2}\Delta P_{E}$. She finally obtained the following:

4.8 $$\Delta P_{E}=\frac{(m^{\prime }+m^{\prime \prime })\alpha_{E}V}{\pi r_{\omega \delta }^{2}\Delta t}=\frac{\rho_{0}(v_{m^{\prime }}+v_{m^{\prime \prime }})\alpha_{E}V}{\pi r_{\omega \delta }^{2}\Delta t}=\frac{\alpha_{E}}{2}\rho_{0}sV.$$

### 4.2. Instantaneous maximum pressure at the instant of liquid droplet impact

The most significant aspect of the analysis by Engel is that she assumed that Δ*t* is *finite*; however, the results obtained by numerical analysis in §3 show that the time elapsed for the pressure on the central axis to reach the maximum pressure converges to 0. Therefore, we should examine whether equation (4.8) is valid even for the instantaneous maximum pressure rise. In other words, we should answer the question: *Is equation (4.8) valid in the limit of* Δ*t to 0, although* Δ*t does not explicitly appear in equation (4.8)?*

First, we discuss the compression process. Engel assumed that the radius of curvature of the droplet in the vicinity of the impact changes instantaneously *with the point impact remaining* in the compression process, as shown in figure 10. We should understand that it takes a finite compression time Δ*t*′ for the liquid in the vicinity of the impact to be compressed, where Δ*t*′ is the time that it takes for the compression wavefront to travel through the compressed region. We now reexamine how the droplet can be compressed in Δ*t*′.

Our numerical results in §3.4 showed that while the intersection advances a distance of 5$\Delta r^{*}(=5\times 2^{-15})$ in the *r*-direction during this compression time of 6$\Delta t^{*}(=2.28\times 10^{-9})$, the solid surface moves by a distance $7.32\times 10^{-5}\Delta r^{*}$ and the compression wavefront travels in the liquid droplet by a distance $1.30\times 10^{-3}\Delta r^{*}$ in the *z*-direction. We are hence considering an extremely small compression time. This consecutive impact of the interface on the solid surface in the surface cells corresponds to the expansion of the circular intersection between the solid surface and the surface of the liquid droplet.

The expansion speed of the circular intersection is much faster than the compression wavefront speed, as Lesser [16] also pointed out. When the circular intersection expansion is caused purely by compression, the circular intersection expansion speed at Δ*t*′ after the impact *V*_*R*_ can be written as

4.9 $$V_{R}=\frac{V(R-V\Delta t^{\prime })}{\sqrt{V\Delta t^{\prime }(2R-V\Delta t^{\prime })}}.$$

Note that *s*/*V*_*R*_ is a good measure to evaluate which process dominates in the compression process, the expansion of the circular intersection or the change in the curvature due to the compression wavefront propagation. We evaluate *s*/*V*_*R*_, taking the limit of Δ*t*′ to 0, obtaining

4.10 $$\lim_{\Delta t^{\prime }\to 0}\frac{s}{V_{R}}=\lim_{\Delta t^{\prime }\to 0}\frac{\sqrt{V(2R-V\Delta t^{\prime })}}{R-V\Delta t^{\prime }}\frac{s}{V}\sqrt{\Delta t^{\prime }}=0.$$

We now understand that the compression deformation in the *z*-direction, as shown in figure 10, never occurs because the compression wavefront should pass through a portion in the liquid droplet for that portion to deform. We conclude that the circular intersection between the solid surface and the surface of the liquid droplet expands at an extremely high speed, much greater than the compression wavefront speed, compressing the spherical cap over which the circular intersection has moved while keeping the radius of curvature of the droplet unchanged in the compression process.

**Figure 11.**
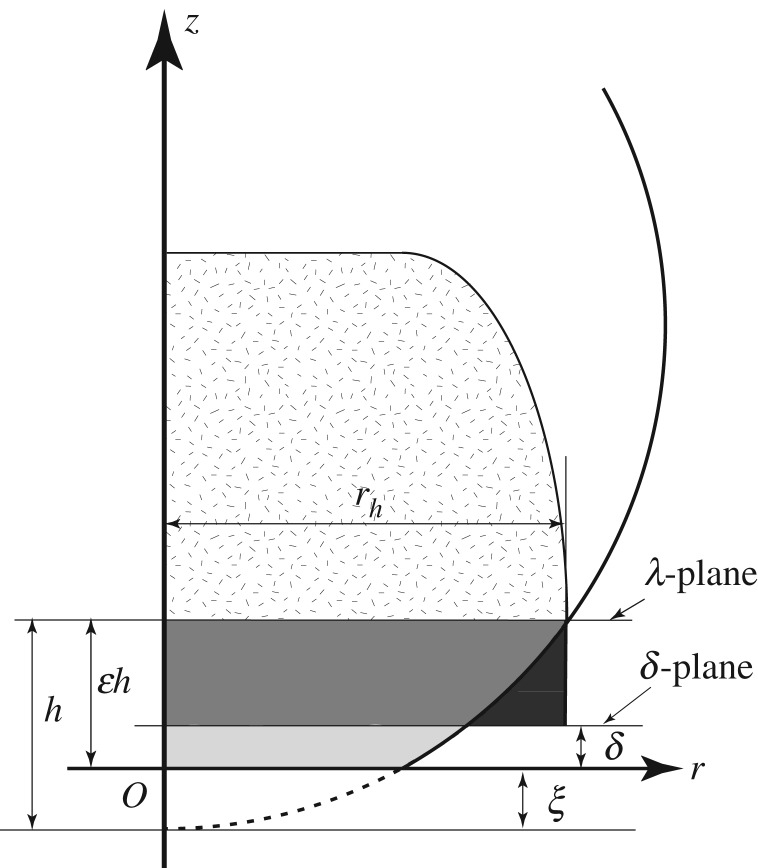
Cross section of a liquid droplet. The original shape of the droplet before compression is indicated by a dotted line. The liquid cylinder with base radius *r*_*ωδ*_ formed between the *δ*- and λ-planes has the same volume as that of the spherical cap whose base is on the λ-plane with height *ωδ*. We consider the limit of *h* to 0 as we take the limit of Δ*t* to 0. Note that the *z*-direction distance travelled by the compression wavefront generated at the compression process is uniform (the straight horizontal line on top of the hatched region) because the circular intersection expansion speed is much faster than the speed of sound. Note that the magnitude of *ξ* is greatly exaggerated here, i.e. *ξ* ≪ *R*.

Our new finding leads to the following. At the end of the droplet compression process, where neither displacement of liquid nor propagation of compression wavelets has occurred, we assume that the equation of the λ-plane is *z* = 0, with no change in the radius of curvature of the droplet in the vicinity of the impact, but a compression of the spherical cap, as shown in figure 11. We set the *z*-coordinate of the first impact point between the solid plane surface and the liquid droplet to be *z* = −*ξ*. We assume that no point contact can be sustained, even at the end of the compression process; hence, the contact area between the liquid droplet and solid surface should be finite at the end of the compression process.

We next discuss the displacement process. After the compression process, the solid plane surface, i.e. the *δ*-plane, moves a distance *δ* and the λ-plane moves a distance of (*h* − *ξ*) over Δ*t*, i.e. the λ-plane reaches *z* = *ɛh* at Δ*t*, as shown in figure 11, where *ɛ* is defined by

4.11 $$\varepsilon =1-\frac{\xi }{h}=1-\nu .$$

As we discussed above, the circular intersection expansion speed is much faster than both the compression wavefront speed and the impact speed, especially at the very early stage of impact; hence, the volume of the compressed spherical cap can also be much larger than the volume of the displaced portion of the liquid droplet, which is indicated by the light grey region in figure 11. Note that *V*/*V*_*R*_ is a good measure for evaluating which process dominates the deformation of the spherical cap, the compression process or the displacement process. We evaluate *V*/*V*_*R*_, taking the limit of Δ*t* to 0 using equation (4.10), obtaining lim _Δ*t*→0_ *V*/*V*_*R*_ = 0. We conclude that *ɛ* converges to 0 in the limit of Δ*t* to 0, i.e. at the instant of the droplet impact, because the compression process completely dominates the deformation of the spherical cap.

Using the notation used in §4.1, we obtain the relation *ωδ* = ε*h*; then, *h* is written as

4.12 $$h=\frac{\omega (1-\alpha_{E})V\Delta t}{\varepsilon }.$$

We now investigate the pressure rise in the limit of Δ*t* to 0. First, we consider the volume *v*′_*m*′_ of *m*′. Recall that *v*_*m*′_ is the volume of a spherical cap with a height of *ωδ* and the radius of the base of *r*_*ωδ*_. *v*′_*m*′_ is the volume of a spherical segment with a radius of the base of *r*_*h*_ and a height of *h*(1 − ν). Simple algebra gives

4.13 $$v_{m^{\prime }}^{\prime }=\frac{\pi }{2}r_{h}^{2}h(1-\nu^{2}).$$

Second, the volume *v*′_*m*″_ of *m*″ is considered. Note that the upper bound of the integration in equation (4.7) is the maximum distance travelled by the compression wave from the λ-plane; hence, *ωβδ* should be replaced by *βh*(1 − ν) in equation (4.7). Then, an evaluation of the integration gives

4.14 $$v_{m^{\prime \prime }}^{\prime }=\frac{\beta \pi }{2}r_{h}^{2}h(1-\nu^{2}).$$

We estimate the average rate of change of momentum of the liquid in volume *v*′, which is

4.15 $$v^{\prime }=v_{m^{\prime }}^{\prime }+v_{m^{\prime \prime }}^{\prime }=\frac{\left(1+\beta )(1-\nu^{2}\right)}{2}\rho_{0}\pi r_{h}^{2}hV,$$

to be (*m*′ + *m*″)*α*_E_*V*/Δ*t*. Noting that instead of equation (4.1), the velocity $V_{\lambda }$ of λ-plane is given by

4.16 $$V_{\lambda }=\frac{\varepsilon h}{\Delta t}=\frac{\epsilon h(1-\alpha_{E})}{\delta }V.$$

We obtain the equation for the instantaneous pressure rise Δ*P*_i_ at the instant of impact:

4.17 $$\Delta P_{i}=\lim_{\varepsilon \to 0}\frac{\rho_{0}v^{\prime }\alpha_{E}V}{\pi r_{h}^{2}\Delta t}=\lim_{\varepsilon \to 0}\frac{\alpha_{E}\rho_{0}(2-\varepsilon )\pi r_{h}^{2}sV\Delta t}{2\pi r_{h}^{2}\Delta t}=\lim_{\varepsilon \to 0}\frac{\left(2-\varepsilon \right)}{2}\alpha_{E}\rho_{0}sV=\alpha_{E}\rho_{0}sV.$$

To obtain these results, we used the fact that *ɛ* converges to 0 in the limit of Δ*t* to 0. If the compression process completely dominates, as in the case we examined, *ɛ* becomes 0. On the contrary, if the displacement process completely dominates, *ɛ* could become 1. When *ɛ* is 1, the coefficient of *ρ*_0_*sV* in equation (4.17) becomes *α*_E_/2, which gives equation (1.1). We found that the coefficient $\frac{1}{2}$ appears when the displacement process completely dominates or the radial flow becomes prominent. Recall that we found in §3.2 that *α* is 0.455 when the liquid in a significantly large Cell-1 can flow. We hence conclude that the coefficient $\frac{1}{2}$ that appears in equation (1.1) originates from the spherical shape of the liquid droplet that can allow the radial flow. We emphasize that *α* can be $\frac{1}{2}$ only when *ɛ* = 1; however, *α* cannot be $\frac{1}{2}$ in the limit of Δ*t* to 0, rather *α* is 1, i.e. *ɛ* is 0. Indeed, *ɛ* can be nothing but 0 at the instant of the droplet impact.

Note that the traversal of the compression wavefront in the droplet may be severely impulsive; hence, the divergence of the compression wave may be negligibly small. Then, we may regard *α*_E_ as 1, which also enforces the point impact because *h* in equation (4.12) converges to 0 in the limit of *α*_E_ to 1. Then, we finally obtain

4.18 $$\Delta P_{i}=\rho_{0}sV.$$

We have successfully extended the equation for the maximum pressure rise Δ*P*_E_ developed at droplet impact given by equation (4.8) in the limit of Δ*t* to 0, to obtain the equation for the instantaneous maximum pressure rise Δ*P*_i_ at the instant of impact given by equation (4.18).

Equation (4.18) is exactly the same as equation (2.11), which predicts the impact pressure rise under the assumption of a one-dimensional flow. Equation (4.10) shows that the central pressure develops much more slowly than the neighbouring contact between the gas–liquid interface at the instant of liquid droplet impact, i.e. in the limit of Δ*t*′ to 0, as we have observed in the numerical results in §3.4; hence, the pressure development on the *z*-axis is forced to be confined to one dimension, as we have discussed in §3.3. Therefore, we conclude that the instantaneous pressure rise due to the spherical liquid impact can be well predicted by equation (4.18).

## 5. Conclusion

The prime objective of this study was to answer how high is the pressure that develops at the instant of a liquid droplet impact on a solid plate. To achieve this objective, we investigated the following issue: *At the instant of impact, is the assumption that the generated pressure coincides with the water-hammer pressure rise relevant?* The water-hammer theory and the shock relation yield the one-dimensional liquid impact pressure rise Δ*P*_s_:

5.1 $$\Delta P_{s}=\rho_{0}sV,$$

where *ρ*_0_ is the density of the undisturbed liquid, *s* is the compression wavefront propagation speed in the disturbed liquid and *V* is the impact velocity. We showed both numerically and analytically that the liquid droplet impact pressure, which is referred to as the pressure developed at the instant of the liquid droplet impact on the contact point, can be predicted by equation (5.1).

We examined the pressure rise at the impact of the liquid droplet by numerically solving two-dimensional axisymmetric compressible Euler equations using the level set ghost fluid method and the stiffened-gas equation of state. We investigated the pressure of the fluid in the surface cells that are adjacent to the solid surface. We defined Cell-1, which is the surface cell that contains the *z*-axis, and the central pressure, which is the pressure in Cell-1. We found that the maximum central pressure rise Δ*P*_max_ depends on the calculation cell size, and it converges to the one-dimensional liquid impact pressure rise Δ*P*_s_ (equation (5.1)) as the cell size decreases. When the non-dimensional calculation cell size $\Delta r^{*}$, which is the dimensional cell size divided by the droplet radius, is 2^−15^, Δ*P*_max_ reaches 0.994Δ*P*_s_. We also found that the elapsed time for the pressure to reach the maximum from the instant of impact $\tau_{e}^{*}$ decreases as $\Delta r^{*}$ decreases and converges to 0.

We found that the maximum central pressure Δ*P*_max_ is greatly affected by the status of Cell-2, which is the surface cell adjacent to Cell-1, while the central pressure increases. When the cell size is sufficiently small, the pressure in Cell-2 begins to rise immediately after the pressure in Cell-1 begins to rise. They rise practically simultaneously. When the fluid in Cell-2 remains a gas after the pressure rises in Cell-1, the liquid in Cell-1 is confined to the space bounded by the gas in Cell-2; hence, the liquid in Cell-1 can be compressed in directions both normal and parallel to the solid surface. In other words, the liquid in Cell-1 can flow in a direction parallel to the solid surface. When the liquid in Cell-1 cannot flow, the flow can be assumed to be one-dimensional. Therefore, Δ*P*_max_ can reach Δ*P*_s_

We then discussed whether the impact pressure rise can be reduced from stiffened-gas pressure rise Δ*P*_sg_ when the interface that impacts on a solid surface has a finite radius of curvature by using the numerical analysis. Numerical calculations were carried out under the conditions $\Delta r^{*}=2^{-15}$ and $\Delta t^{*}=3.73\times 10^{-10}$. Under these conditions, we successfully reproduced the droplet impacts with a solid surface with a finite radius curvature because no simultaneous interface impacts occurred in any adjacent cells. The maximum central pressure rise Δ*P*_max_ was 0.994Δ*P*_s_. We hence concluded that the maximum central pressure rise that develops when a droplet impacts on a solid surface can be accurately predicted by equation (5.1), *even with a finite radius of curvature interface*. We emphasize that it requires extremely high resolution in both the spatial and temporal domains to obtain the numerical result that the maximum central pressure rise can be predicted by equation (2.10). We are convinced that insufficient resolutions in either the numerical or experimental studies caused the previous confusion in determining the factor.

We shed light on the prediction of the maximum pressure rise Δ*P*_E_ by [6],

5.2 $$\Delta P_{E}=\frac{\alpha_{E}}{2}\rho_{0}sV,$$

where, for comparison, the compression wavefront propagation speed *s* is used instead of *c*_0_, the sound velocity in the undisturbed liquid that was used in the original equation. It is widely recognized that *α*_E_/2 comes from the spherical shape of the liquid droplet; however, our numerical analysis showed that the impact pressure rise due to the spherical liquid droplet can be predicted by equation (5.1), where no shape factor is required. We discussed the origin of the mysterious factor *α*_E_/2; then, revealed the paradox.

We realized that Engel [6] derived equation (5.2) for the maximum pressure rise developed at finite time Δ*t* after a liquid droplet impacts on a solid surface, not for the maximum pressure rise at the instant of impact, and that she did not take into account that the progression speed of the contact between the gas–liquid interface and the solid surface is much faster than the compression wavefront propagation speed at the instant of the impact. We corrected Engel’s elastic impact model taking the limit of *δt* to 0 by carefully examining the compression of the cap of the spherical liquid droplet at the compression process at the instant of impact. We found that the factor *α*_E_/2 appearing in equation (5.2) converges to 1, at the instant of impact in the limit of impact duration time Δ*t* to 0, because the central pressure develops much more slowly than the neighbouring contact progresses between the gas–liquid interface and the solid surface. We conclude that we successfully proved that the corrected Engel’s theory can also provide the maximum central pressure rise Δ*P*_s_, equation (5.1), at the instant of impact between a spherical liquid droplet which keeps its spherical shape at the instant of the impact and a rigid solid surface.

## Supplementary Material

How the pressure rise time depends on calculation cell size

## Data accessibility

Our codes, the output data and the data used to generate the figures in this work are publicly available at https://github.com/MasaoWatanabe/Liquid-Droplet-Impact-Pressure.
