# Supplementary material for: Pressure generated at the instant of impact between a liquid droplet and solid surface
Source: R Soc Open Sci. 2018 Dec 12;5(12):181101. doi: 10.1098/rsos.181101 (PMC6304138; doi:10.1098/rsos.181101)
Supplement: How the pressure rise time depends on calculation cell size [file rsos181101supp1.pdf]

ROYAL SOCIETY OPEN SCIENCE

rsos.royalsocietypublishing.org

Research

Check for updates

Subject Areas:
mechanical engineering, fluid mechanics, computational mechanics

Keywords:
droplet impact, impact pressure, water-hammer pressure, shock wave, shape factor

Author for correspondence:
M. Watanabe
e-mail: masao.watanabe@eng.hokudai.ac.jp

Supplement: Pressure generated at the instant of impact between a liquid droplet and solid surface

Y. Tatekura[1], M. Watanabe[1], K. Kobayashi[1] and T. Sanada[2]

[1]Graduate School of Engineering, Hokkaido University, Kita 13, Nishi 8, Kita-ku, Sapporo Japan
[2]Graduate School of Engineering, Shizuoka University, 3-5-1 Johoku, Naka-ku, Hamamatsu, Japan

S1. How the pressure rise time depends on calculation cell size

As we have considered in Section 3, it takes a finite time for the pressure in Cell-1 to reach the maximum pressure after the instant of impact. Figure 5 in Section 3(b) shows that the nondimensional elapsed time τ_e^* decreases as the nondimensional cell size Δr^* decreases. We now discuss how τ_e^* depends on Δr^* in detail.

At the instant of impact ($t = 0$), the fluid in the Cell-1 turns from gas to liquid. We refer to the liquid that initially occupies Cell-1 (with height of L and the cross-sectional area A) as the object liquid. Figure S1.1(a) shows the object liquid at $t = 0$. The volume of the object liquid is the same as that of Cell-1 at $t = 0$. Here, the liquid is elastic; hence, it is compressed as the pressure rises. When Δr^* is sufficiently small, ϕ becomes also too small for the liquid in Cell-1 to flow as discussed in Section 3(c). We can then assume that the object liquid deforms only in the direction normal to the solid surface and the cross-sectional area A is kept constant.

In the numerical analysis, only one value for each physical property can be defined in Cell-1; hence, we proceed with our analysis in the same manner. We assume that only one value for velocity V, pressure p_0, and density ρ_0 is assigned to the object liquid at $t = 0$. The velocity in the z-direction of the object liquid is V at $t = 0$ with a zero-velocity condition imposed on the solid

THE ROYAL SOCIETY PUBLISHING

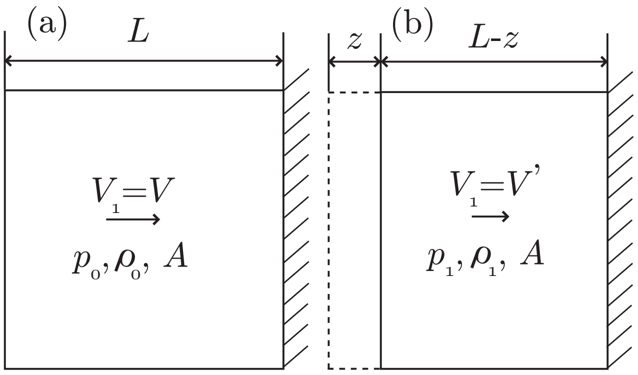

Figure S1.1. Deformation of fluid in Cell-1. At the instant of impact ($t=0$), the fluid in Cell-1 turns from gas to liquid. We refer to the liquid that initially occupies Cell-1 (with height of L and the cross-sectional area A) as the object liquid. The object liquid at $t=0$ is shown in (a). The volume of the object liquid is the same as that of Cell-1 at $t=0$. Here, the liquid is elastic; hence, it is compressed as the pressure rises. After time t passed, the height of the object liquid is reduced from L to $L-z$ with velocity V', pressure p, and density ρ, as shown in (b). The infinitesimal compression dz of the object liquid for the infinitesimal time dt at t can be written as $dz = V'dt$.

surface; then, both the height and velocity of the object liquid gradually decrease as time progresses. After time t passed, the height of the object liquid is reduced from L to $(L-z)$ with velocity V', pressure p, and density ρ, as shown in figure S1.1(b). The infinitesimal compression dz of the object liquid for the infinitesimal time dt at t can be written as

$$dz = V'dt. \tag{S1.1}$$

The mass of the object liquid is conserved during the deformation; hence, the conservation of mass holds:

$$\frac{L-z}{L} = \frac{\rho_0}{\rho}. \tag{S1.2}$$

We used two-dimensional axisymmetric compressible Euler equations for an ideal fluid, Eqs. (2.1), (2.2), and (2.3), in our numerical analysis. Hence, we consider the isentropic compression of the object liquid in the present analysis using the stiffened-gas equation of state (2.8),

$$\frac{\rho_0}{\rho} = \left(\frac{p_0+\Pi}{p+\Pi}\right)^{1/\gamma}. \tag{S1.3}$$

Equations (S1.1), (S1.2), and (S1.3) yield the pressure rise rate of the object liquid:

$$\frac{dp}{dt} = \frac{\gamma(p+\Pi)}{L}\left(\frac{p+\Pi}{p_0+\Pi}\right)^{1/\gamma} V'. \tag{S1.4}$$

We consider the one-dimensional shock wavefront propagation in the liquid after droplet impact with an impact velocity V of 100 m/s, which causes an abrupt pressure rise in the liquid as discussed in Secion 2. We assume uniform physical properties in the portion of liquid through which the compression wave has propagated as shown in figure S1.2. Previously, we considered the case in which the velocity in the disturbed liquid is zero (figure 1 in Section 2) when the water-hammer pressure rise was discussed. We now consider the case in which the disturbed liquid has non-zero velocity V' as shown in figure S1.3(b). The pressure, density, and velocity of the undisturbed liquid are p_0, ρ_0, and V, respectively, and those of the disturbed liquid are p, ρ, and V', respectively. The compression wavefront speed is s. We use the coordinate system moving with the speed of $s-V$.

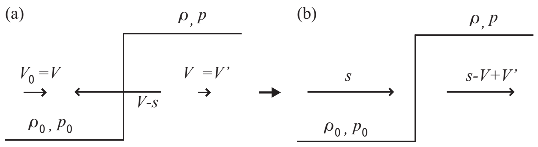

Figure S1.2. Compression wavefront propagation into an undisturbed fluid. We consider the one-dimensional shock wavefront propagation in the liquid after droplet impact with the impact velocity V of 100 m/s, which causes an abrupt pressure rise in the liquid, as discussed in Section 2. We assume uniform physical properties in the portion of liquid through which the compression wave has propagated.

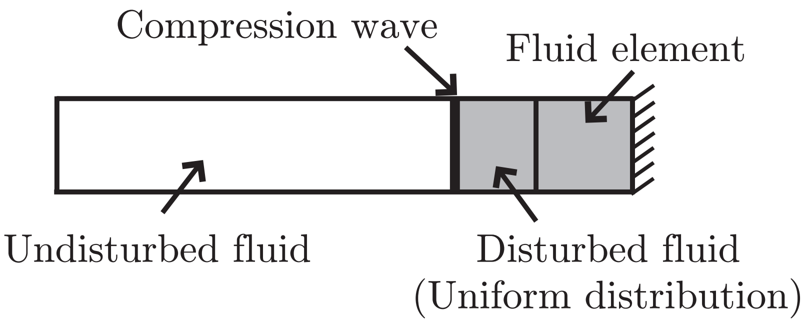

Figure S1.3. Properties in front of and behind the shock with disturbed fluid velocity V' (a) in the stationary coordinate system and (b) in the moving coordinate system with velocity $(s - V + V')$.

Equations (2.1), (2.2), and (2.3) lead to

$$\rho_0 s = \rho[s - (V - V')], \tag{S1.5}$$

$$p_0 + \rho_0 s^2 = p + \rho[s - (V - V')]^2, \tag{S1.6}$$

$$p - p_0 = \rho_0 s(V - V'). \tag{S1.7}$$

The relation of the pressure and the velocity of the object liquid can be written as

$$V' = V - \frac{p - p_0}{\rho_0 s}. \tag{S1.8}$$

Substituting Eq. (S1.8) into Eq. (S1.4), we obtain a differential equation for the pressure rise rate:

$$\frac{dp}{dt} = \gamma(p + \Pi)\left(\frac{p + \Pi}{p_0 + \Pi}\right)^{\frac{1}{\gamma}}\left(V - \frac{p - p_0}{\rho_0 s}\right)\frac{1}{L}. \tag{S1.9}$$

We compare the pressure development obtained by the time integration of Eq. (S1.9) using the Euler method with that obtained by the numerical simulation. The results are shown in figure S1.4, where nondimensionalized time t^* is defined by $t^* = Vt/R$ as in figure 4 in Section 3(b). Smaller Δr^* leads to markedly better agreement between the numerical results and the prediction obtained by Eq. (S1.9).

We now estimate the time t_p that it takes for the pressure to rise from p_0 to p at the very early stage of impact. Note that sound velocity s can be written as

$$s = c_0\sqrt{\frac{\gamma + 1}{2\gamma}\left(\frac{p}{p_0} - 1\right)\frac{p_0}{p_0 + \Pi} + 1}, \tag{S1.10}$$

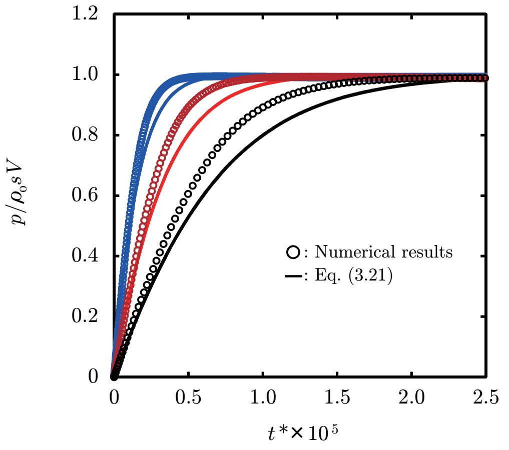

Figure S1.4. Comparison of the pressure development obtained by a time integration of Eq. (S1.9) using the Euler method with that obtained by the numerical simulation. Black, red, and blue open circles are the numerical results with $\varDelta r^*$ of 2^{-13}, 2^{-14}, and 2^{-15}, respectively. Black, red, and blue lines are the corresponding results obtained by Eq. (S1.9). Nondimensionalized time t^* is defined by $t^* = Vt/R$ as in figure 4 in Section 3(b). Smaller $\varDelta r^*$ leads to markedly better agreement between the numerical results and the prediction obtained by Eq. (S1.9).

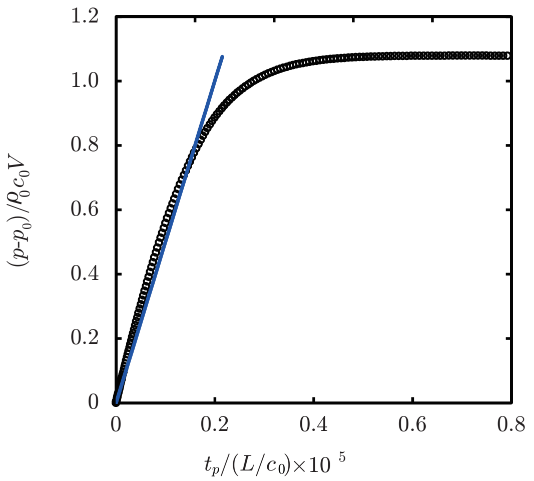

Figure S1.5. Pressure rise obtained from Eq. (S1.12) at Cell-1 with an impact velocity of $100\ \mathrm{m/s}$ (blue line) compared with the numerical result (black open circles) for $\varDelta r^* = 2^{-15}$. Equation (S1.12) can predict the pressure rise at the very early stage of the impact well. We define the characteristic time t_c of the pressure rise at the very early stage of the impact as follows: $t_c = L/c_0$.

using the stiffened-gas equation of state (2.8). We can assume that p can be well approximated by p_0 at the very early stage of impact; then, Eq. (S1.9) can be simplified to

$$\frac{dp}{dt} = \gamma(p_0 + \varPi)\frac{V}{L}. \tag{S1.11}$$

We Integrate Eq. (S1.11), noting that the condition $p = p_0$ leads to $s = c_0$ in Eq. (S1.10), yielding

$$\frac{p - p_0}{\rho_0 c_0 V} = \frac{t_p}{L/c_0}, \tag{S1.12}$$

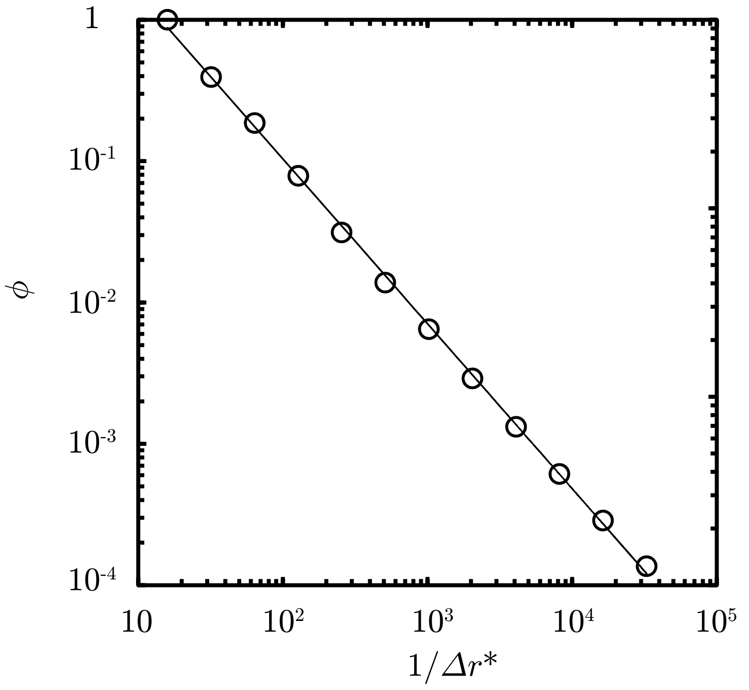

Figure S1.6. The dependency of ϕ (defined by Eq. (3.3)) on Δr^*. ϕ (in log-scale) is plotted on the ordinate and Δr^* (in log-scale) is plotted on the abscissa. The regression line shows that ϕ is approximately proportional to Δr^*; ϕ is proportional to the power of 1.167.

where

$$c_0 = \sqrt{\frac{\gamma(p_0 + \Pi)}{\rho_0}}, \tag{S1.13}$$

is used. Figure S1.5 shows that Eq. (S1.12) can well predict the pressure rise at the very early stage of the impact. We define the characteristic time t_c of the pressure rise at the very early stage of impact as follows: $t_c = L/c_0$.

Equation (S1.12) shows that it takes a finite time for the pressure to rise at the very early stage of impact because a pressure wave with a velocity of c_0 should propagate through the finite height L of the calculation cell in the numerical analysis. Equation (S1.11) also shows that the pressure rise rate of the object liquid is inversely proportional to cell height L. Noting that ρ_0, p_0, s, V, γ, and Π are constants, It is clear that the time required for the impact pressure to reach the maximum decreases as L decreases in the numerical analysis, as shown in figure S1.5. These results suggest that impact pressure should rise instantly at the impactas $L \to 0$.

S2. Comments on the experimental measurements

Let us briefly explain the difficulties in experimental measurement of the pressure generated at the droplet impact. We start from the Huyghens principle that can well predict the profile of the compressed wavefront as shown by Lesser [1]. He used the Huygens principle that at each instant the expanding contact line emits a wavelet propagating with the speed s. The individual wavelets are emitted by the expanding contact line. During the initial stages of the droplet impact, the expanding speed of the contact line can be written as $(R - V\tau)/\sqrt{2RV\tau - V^2\tau^2}$, where R is droplet radius, V is impact velocity, τ is elapsed time from the instant of the impact. It can be

easily shown that the expanding speed of the contact line is extremely greater than the speed of sound in the liquid when $\tau \simeq 0$. Then, the contact line extends not because the liquid mass in the vicinity of the contact line extrudes to form a thin liquid layer, but because the liquid mass on the droplet surface impacts consecutively onto the solid surface. These consecutive impacts causes the impact pressure at the contact line $\boldsymbol{c}(\tau)$.

By the Huyghens principle, at each instant the extending contact line will emit a wavelet moving at the compression wavefront speed s [1]. The envelope of the individual wavelets forms a compression wave front separating a disturbed liquid region from an undisturbed liquid region. The envelope of these wavelets consists a compression wavefront. Lesser derived the critical time τ_c when the expanding speed of the contact line is equal to the compression wavefront speed s. When $\tau < \tau_c$, compression wavefront cannot overtake the contact line, but is pinned at the contact line. The geometrical acoustics model proposed by Lesser [1] enabled us to evaluate τ_c, which can be defined as

$$\tau_c = M_i R/(2s), \tag{S2.1}$$

and c_{r0}, which is the horizontal coordinated of the contact line when the expanding speed of the contact line is equal to s:

$$c_{r0} = M_i R. \tag{S2.2}$$

Suppose we aim to measure the pressure generated at the water impact of droplet experimentally. The typical size of droplet used in the droplet impact experiments is 1 mm. The impact velocity $V = 100$ m/s and $c_0 = 1,625$ m/s is used to reproduce our numerical results shown in our manuscript; hence $M_i = 0.06$. Note that s in Eq. (S2.1) is greater than $s = 1,760$ m/s because the pressure at the critical point is greater than the pressure at the instant of the impact; however, we use this value to approximately evaluate τ_c, which may give us larger τ_c than the accurate evaluation. Substituting these values in Eqs. (S2.1) and (S2.2), we obtain that $\tau_c \simeq 18$ ns, and $c_{r0} \simeq 60$ μm. This simple evaluation suggests that we should carry out experiments with extremely ultra-high resolutions in both space and time to verify our results.

Furthermore, the pressure generated at the impact is never constant. Heymann [2] predicted the pressure at the critical point. He found that the pressure generated at the critical point where the compression wavefront detach the contact point is significantly greater (approximately 2 to more than 10 times greater depending on the impact Mach number M_i) than the pressure generated at the instant of the impact. This spatial distribution of the pressure generated on the solid surface makes us much harder to carry our experimental measurements of the pressure on the point of the contact at the instant of the droplet impact.

To the best of our knowledge, the pressure measurement in the region beneath the impacting liquid droplets with the highest resolutions in both space and time were carried out by Bourne [3]. He measured the impacting pressure of droplets onto polymethylmethacrylate (PMMA) by using polyvinylidene difluoride (PVDF) stress gauge. Although the rise time of the PVDF was approximately 100 ns, which is rather longer than that of the managing gauge (20 - 30 ns), the spatial resolution of his experiment was 25 - 30 μm along the surface of the target. He measured the pressure at the impact of a spherical water droplet with the radius of 2 mm with the velocity of 600 m/s. He found that the value of the initial pressure along the central axis was approximately 0.8 GPa. Now, we consider whether his result verifies our results.

First, we calculate τ_c and c_{r0} by using Eqs. (S2.1) and (S2.2) for $M_i = 0.37$ and $R = 2$ mm. We obtain that $\tau_c \simeq 220$ ns, and $c_{r0} \simeq 720$ μm. Although the spatial resolution (25 - 30 μm) of Bourne's experiment is of the order of one-tenth of c_{r0}, the rise time ($\sim$100 ns) is the same order of τ_c. We must consider that the resolution, especially temporal resolution, of even Bourne's experiment is not high enough to validate the results of our paper. We, then, consider the value of the initial pressure (0.8 GPa) that Bourne obtained. We can evaluate the pressure by using the classical water-hammer theory (Eq. (1.2)). The estimated pressure is 0.975 GPa; however, the more accurate evaluation of the pressure by using Eq. (1.3) gives 1.57 GPa (with Eq. (2.9)) or 1.69 GPa (with

Eq. (2.7)). These values are approximately twice greater than the experimentally obtained value by Bourne. We consider that the primary reason for the discrepnacy is that the PMMA is not a rigid but rather elastic material. For impact on a compliant target, the classical water-hammer pressure is reduced to P_c [4]:

$$P_c = \rho s \rho_s s_s V / (\rho s + \rho_s s_s) = \rho s V/(Y+1), \tag{S2.3}$$

where ρs and $\rho_s s_s$ are the acoustic impedance of water and PMMA, respectively, and Y is the ratio of the acoustic impedance:

$$Y = (\rho s)/(\rho_s s_s). \tag{S2.4}$$

We approximate Eq. (S2.4) by $Y = (\rho c_0)/(\rho_s c_{0s})$ due to the lack of knowledge in the accurate equation of state for PMMA, where $c_0 = 1625$ m/s and $c_{0s} = 2746$ m/s are the speed of sound in an undisturbed liquid of water and PMMA, respectively. Then, we obtain $Y = 0.50$ and $P_c = 1.05$ GPa. This value is still greater than the value that Bourne obtained; however, not twice greater. Bourne also admitted in his paper that the pressure may be underestimated because of sampling frequency. As we have reviewed, more accurate experimental results with much higher resolutions in both space and time are eagerly awaited, although the experimental results by Bourne and others suggested the unnecessity of the shape factor.

S3. The effect of finite-size calculation cell

We analyzed the pressure generation at droplet impact by solving the two-dimensional axisymmetric compressible Euler equations [5,6] within a framework of continuous mathematics in a sense that physical properties such as velocity, pressure, and entropy are defined as continuous functions of time and space. However, it is well known that Euler equations also allow non-continuous solutions such as shock waves as weak solutions. In other words, Euler equations can have series of continuous solutions that converge to the solutions that have discontinuities on a null set in a sense of Lebesgue measure, i.e., weak convergence. Although these solutions are weak solutions, they are in general considered as the solutions obtained from the continuum theory within a framework of continuous mathematics without any discussion of molecular scale.

Our objective of this study is to evaluate the pressure generated on a contacting point between a solid surface and a liquid surface with a finite curvature at the instant of the impact to discuss the spherical factor proposed by Engel [7]. We investigated the pressure generated on a point at the instance of the impact. Though the Lebesgue measure of a point is zero, we still can evaluate values at the point within a framework of continuous mathematics by taking limit of the measure to be zero, or the time to be zero.

We used finite difference method, i.e., discrete mathematics, to find the solution of Euler equations. We found the numerically calculated value converges to a value with the decrease of the calculation grid size, ultimately in the limit of zero. Therefore, we concluded that our numerically calculated value is surely a solution of Euler equations within a framework of continuous mathematics.

We recognize that the question if the mathematically obtained solution can explain the physical phenomena should be answered; however, to answer this question, we need either experimental results with extremely ultra-high resolutions in both space and time, or numerical results of non-equilibrium molecular dynamics with extremely ultra-large scale. These are beyond the scope of this study. Nevertheless, we succeeded to provide the answer for the discussion regarding to the spherical factor proposed by Engel within a framework of continuous mathematics.

We discussed how the pressure rise time depends on calculation cell size in Section S1. We explained there that it takes a finite time of the pressure of a cell to reach the maximum pressure, and that the rise time. We showed that the important parameter ϕ defined by Eq. (3.3) is linearly proportional to the cell size. In figure 7 in Section 3(c), we also found that $(1-\alpha)$ converges to 0 with approximately proportional to the power $(1/2)$ of ϕ, or to the power $(1/2)$ of the cell size.

Hence, convergence itself is not significantly slow. We consider that the reason why it required significantly small size of calculation cell is that we have to obtain a value at a point.

References

1. Lesser MB. 1981 Analytic solutions of liquid-drop impact problems. *Proc. R. Soc. Lond A Math. Phys. Sci.* **377** (1770), 289-308. (doi: 10.1098/rspa.1981.0125)
2. Heymann FJ. 1969 High-speed impact between a liquid drop and a slid surface. *J. Appl. Phys.* **40** (13), 5113-5122. (doi: 10.1063/1.1657361)
3. Bourne NK. 2005 On impacting liquid jets and drops onto polymethylmethacrylate targets. *Proc. R. Soc. A* **461**, 1129-1145. (doi:10.1098/rspa.2004.1440)
4. Field JE, Camus JJ, Tinguely M, Obreschkow D, Farhat M. 2012 Cavitation in impacted drops and jets and the effect on erosion damage thresholds. *Wear* **290-291**, 154-160. (doi: 10.1016/j.wear.2012.03.006)
5. Haller KK, Ventikos Y, Poulikakos D, Monkewitz P. 2002 Computational study of high-speed liquid droplet impact. *J. Appl. Phys.* **92** (5), 2821-2828. (doi: 10.1063/1.1495533)
6. Tatekura Y, Fujikawa T, Jinbo Y, Sanada T, Kobayashi K, Watanabe M. 2015 Observation of water-droplet impacts with velocities of O (10 m/s) and subsequent flow field. *ECS J. Solid State Sci. Techol.* **4** (9), N117-N123. (doi: 10.1149/2.0111509jss)
7. Engel OG. 1955 Waterdrop collision with solid surfaces. *J. Res. Natl. Bur. Standa.* **55** (5), 281-298.
